# Development and application of random forest regression soft sensor model for treating domestic wastewater in a sequencing batch reactor

**DOI:** 10.1038/s41598-023-36333-8

**Published:** 2023-06-05

**Authors:** Qiu Cheng, Zhan Chunhong, Li Qianglin

**Affiliations:** 1 Department of Material and Environmental Engineering, Chengdu Technological University, Chengdu, China; 2 Huicai Environmental Technology Co., Ltd., De Yuan Zhen, Pidu District, Chengdu, Sichuan China

**Keywords:** Sustainability, Pollution remediation

## Abstract

Small-scale distributed water treatment equipment such as sequencing batch reactor (SBR) is widely used in the field of rural domestic sewage treatment because of its advantages of rapid installation and construction, low operation cost and strong adaptability. However, due to the characteristics of non-linearity and hysteresis in SBR process, it is difficult to construct the simulation model of wastewater treatment. In this study, a methodology was developed using artificial intelligence and automatic control system that can save energy corresponding to reduce carbon emissions. The methodology leverages random forest model to determine a suitable soft sensor for the prediction of COD trends. This study uses pH and temperature sensors as premises for COD sensors. In the proposed method, data were pre-processed into 12 input variables and top 7 variables were selected as the variables of the optimized model. Cycle ended by the artificial intelligence and automatic control system instead of by fixed time control that was an uncontrolled scenario. In 12 test cases, percentage of COD removal is about 91. 075% while 24. 25% time or energy was saved from an average perspective. This proposed soft sensor selection methodology can be applied in field of rural domestic sewage treatment with advantages of time and energy saving. Time-saving results in increasing treatment capacity and energy-saving represents low carbon technology. The proposed methodology provides a framework for investigating ways to reduce costs associated with data collection by replacing costly and unreliable sensors with affordable and reliable alternatives. By adopting this approach, energy conservation can be maintained while meeting emission standards.

## Introduction

Rural domestic sewage is characterized by unstable water quality and quantity, dispersed discharge and low pollutant concentration^1^. To address these challenges, small-scale distributed water treatment equipment has become widely used in the field of rural domestic sewage treatment due to its rapid installation and construction, low operation cost, and strong adaptability^2^. In recent years, the sequencing batch reactor (SBR) process has emerged as a promising option for rural domestic wastewater treatment. When compared with other processes, SBR can effectively withstand organic load impacts, has flexible operation modes, produces good effluent effects, and achieves better nitrogen and phosphorus removal effects^3–6^.

However, constructing accurate simulation models for rural domestic wastewater treatment can be challenging due to the non-linearity and hysteresis characteristics exhibited by the SBR process^7,8^. The non-linear problems in sewage treatment refer to the complex, diverse, and non-linear relationships that arise from the interactions of various chemical reactions, biological reactions, and physical effects during sewage treatment.

Artificial intelligence, including machine learning, has been applied to sewage treatment processes to effectively solve non-linear problems. Machine learning encompasses a range of methods, such as neural networks and support vector regression, which can be used to analyze and model the complex data generated during sewage treatment. This has effectively improved sewage treatment efficiency and quality while reducing treatment costs.

Artificial neural network (ANN) is a mathematical model that simulates the behavior of animal neural networks, and performs distributed and parallel information processing. ANN has become widely used in predicting sewage discharge, as it can adjust the interconnections among a large number of internal nodes to process complex information within the system^9–13^.

In addition to using artificial neural network (ANN) methods, other techniques such as linear regression (LR), support vector regression (SVR), and neuro-fuzzy network methods have also been used in pollutant removal technology to predict changes in pollutant concentrations or other process parameters^14–19^. These methods (as shown in Table 1) have been proven effective in modeling the complex relationships between various factors and predicting pollutant concentrations, which helps to optimize the performance of the treatment process.

**Table 1:** Methods used in pollutant removal technology to predict changes.

References	Variables/inputs	Targets/outputs	Model performance	Model
^1,4^	pH, time, Initial concentration of Cu(II), Nano zero-valent aluminum dose, stirring rate, and temperature	Cu(II) removal efficiency	MSE:ANN ˂ 10^−5^ LR 0.01 SVR 10^−3^	ANN, LR, SVR
^15^	Temperature, pH, dissolved oxygen (DO), electrical conductivity (EC), NO_3_^−^, and PO_4_^3-^	Dry cell weight	Determination coefficient (R^2^) 0.983	ANN
^16^	Current intensity (I), pH, Fe^2+^ amount, and initial diazinon concentration	Diazinon removal Efficiency	R^2^: 0.994	ANN
^17^	Temperature, pH, time, Initial concentration of Cr(VI), and polyamine/folic acid composite dose	Cr(VI) removal efficiency	R^2^: 0.919	ANN
^18^	pH, conductivity, BOD, COD, TN influents	BOD, COD, and TN effluents	R^2^: BOD 0.764–0.783, COD 0.926, 0.939, TN 0.941–0.957	Neuro-fuzzy networks
^19^	Ten attributes of filament bacteria	SVI	R^2^: 0.78, MSE:6	ANN

However, despite these models^14–17^ performed quite well, their processing or environment is idealized. Most of them use simulated experimental conditions. Once in a real engineering case, due to its complexity, the model's performance will not be so outstanding^18,19^. In addition, in these cases^14–19^, there are many types of input data, such as DO, pH, conductivity, BOD, COD, TN, etc., which increases the workload or difficulty of data acquisition. For example, there is a significant lag in the measured data of DO sensors; BOD can only be measured using biochemical method and cannot be accurately measured online using sensors due to significant hysteresis. However, although COD measurement can be conducted online, the chemical online measurement method requires strict control of measurement conditions and continuous addition of reagents. The COD sensor method mainly uses optical sensors, which are significantly affected by the chromaticity and turbidity of wastewater. Moreover, the COD sensor is expensive for dispersed small equipment, making it difficult to popularize. Therefore, it is necessary to develop sensors with stable and accurate data collection, long service life and cheap price to replace sensors with poor stability, short service life and expensive prices.

However, the traditional ANN algorithm is based on the asymptotic theory, the empirical risk approaches the actual risk only when the sample size approaches infinity, so the sample size is far from infinity in practical application, it leads to the problems of poor extrapolation ability, slow convergence speed and local extremum^20–23^.

Random forest model is one of machine learning, that has become one of research hotspot in the field of artificial intelligence, which has strong adaptive learning ability and nonlinear mapping ability^24,25^. It is suitable for the simulation of wastewater treatment process with the characteristics of large lag, non-linearity and multi-variable^26,27^.

The random forest regression (RFR) is a critical application of the random forest (RF) algorithm, which is a statistical learning theory developed by Breiman^28^. The RFR technique involves using Bootstrap resampling to extract multiple samples from the original data and construct decision trees for each Bootstrap sample. These decision trees are then combined to predict the results, with the final prediction being the average of the predictions generated by all the trees^29^.

The essence of the RFR algorithm is multi-decision tree model, which makes prediction by combining multiple decision trees. The algorithm has the advantages of high prediction precision, good generalization ability, fast convergence speed and less adjustment parameters, which can effectively avoid “over-fitting” and is suitable for the operation of various data sets. It is robust to the variable extraction of data sets and suitable for ultra-high-dimensional variable vector space. RFR has been widely used in many fields such as medicine, management and agriculture^30–32^.

RFR also makes full use of limited samples and construct multiple decision tree models, which increases the diversity of decision tree and improves the accuracy of the final optimization integration model^33,34^. Table 2 shows related applications of random forest regression.

**Table 2:** Application of random forest regression (RFR).

References	Variables/inputs	Targets/outputs	Determination coefficient (r^2^)
^35^	Temperature, precipitation, and wind	PM_2.5_ of Yangtze River Delta of China from 2015 to 2020	> 0.9
^3,6^	County-level census data, natural suitability, and socio-economic factors	population distribution of the Tuojiang River Basin from 1911 to 2010	0.84
^3,7^	Population, agricultural discharge, domestic discharge, sewage collection and treatment way	COD_Mn_ for the Taihu Lake basin in Zhejiang Province, China	0.78
^3,8^	Runoff data in the same month of the first three years and the runoff data of the first three months	Runoff data of river in Xiaojin County, China	0.85
^3,9^	Conductivity, turbidity	Nitrate (89%) Total N (85%) Total P (74%) of the lake george drainage basin of U.S	Nash–Sutcliffe efficiency coefficient (NSE) similarly to the coefficient of determination
^40^	Season, outdoor PM_2.5_ concentration, the number of air cleaners deployed, and the density of gers (traditional felt-lined yurts) surrounding the apartments	Indoor PM_2.5_ concentrations	0.815
^41^	Nitrogen application, agricultural and developed land area, and impervious or developed land in the 100-m stream buffer	Loads of total nitrogen	0.76
^42^	Particulate matter 2.5, soil moisture, and relative humidity	Negative air ion in a warm-temperate region of China	0.931
^43^	Real-time color attributes and the environmental conditions of drying process	Moisture ratio of drying date fruit chips	0.976
^44^	Temperature, Wind speed, relative humidity	Ozone concentration in Malaysia	0.970

ANN is a kind of machine learning algorithm that is commonly used for predicting the treatment effect of sewage water^45–49^. However, one of the major weaknesses of ANN is overfitting, which can lead to a reduction in the model's generalizability^50–52^. In contrast, the random forest regression (RFR) model is another machine learning algorithm used for predicting sewage water treatment effects. The RFR model has several advantages, including high prediction accuracy, fast processing efficiency, strong generalization ability and is not easily susceptible to overfitting^53,54^. These features make the RFR model an attractive option for predicting sewage water treatment effects.

Scholars used RFR to predict pollutants concentration in the ambient air^55–59^ and urban sewage treatment effect^60–62^. However, there are comparatively fewer studies on the prediction and control of rural domestic sewage treatment effects using the RFR model.

The proposed methodology aims to achieve improved prediction and effective control of the treatment effect of rural domestic sewage through the development and utilization of RFR soft sensor model. By utilizing this approach, it is hoped to establish a reliable and robust soft sensor model that can accurately monitor and analyze key indicators of sewage treatment in rural areas. This will not only facilitate the identification of potential issues and assist in their resolution but also contribute to overall improvements in local ecological conditions and public health standards.

Soft sensor is a commonly used method in process monitoring and control, which estimates the process variable of interest based on the measurements of other variables that are easy to acquire. The establishment of a soft sensor model usually involves selecting relevant input variables, designing the mathematical model, and training the model using historical data. The resulting model can then be used for real-time prediction and control. Soft sensors have been widely applied in various industrial processes such as chemical processes, wastewater treatment and power plants. The advantages of soft sensor include cost-effectiveness, flexibility and ability to handle complex nonlinear systems. Soft sensor has proven to be a valuable tool for process optimization and control^63–66^.

## Methods

### RFR model

#### Construction of RFR model

RFR model is an integration algorithm developed on the basis of decision tree theory, which belongs to bagging type^67^. By combining multiple weak learner cart trees and taking the mean value to integrate multiple models, the final result is obtained^68^.

**Figure 1:**
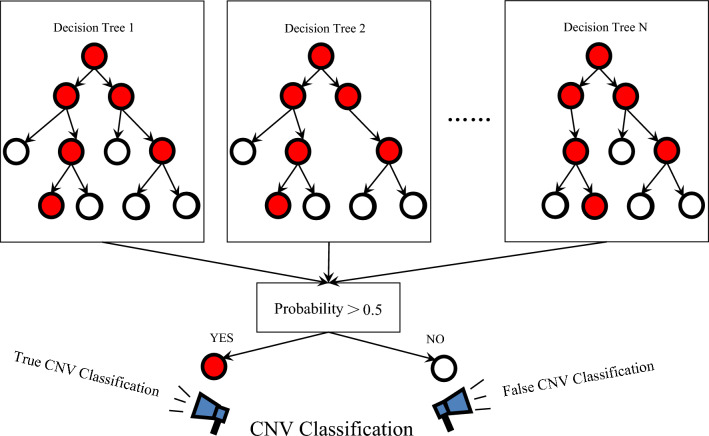
Diagram of RFR model.

The RFR model uses the disturbance of samples and attributes, and increases the "diversity" of the cart tree of the weak learner, so that the final integration result has high accuracy and generalization performance^69^. The RFR model solves practical problems such as small samples, high dimensions and multi-classification, and can handle both discrete data and continuous data^70^. It overcomes the shortcomings of slow convergence speed of neural networks and requires a large number of samples, It also solves the problem of over fitting or under fitting of decision tree, and has good applicability and popularization^71^. Figure 1 shows the diagram of RFR.

#### Prediction method

The general prediction method of RFR model is:

(1) Randomly take samples from training samples (n × sample) for n times to form a training set (samples were put back after every sampling). Repeat r times to obtain training sets:$D_{1} ,D_{2} , \ldots ,D_{r}$.

(2) For each training set, k attributes are randomly selected from the attribute set (m × attribute), $k = \log 2m$, and then cart trees are established:$f_{1} (x),f_{2} (x), \ldots ,f_{r} (x)$.

(3) The final prediction value of random forest is determined by the average method:$f(x) = \frac{1}{r}\sum\nolimits_{i = 1}^{r} {f_{i} (x)}$.

#### Evaluation index of the model

In order to evaluate the performance of the COD concentration prediction model, mean square error (MSE) and determination coefficient (r^2^) are selected as evaluation indexes. The indicators are calculated as follows:

$$ MSE = \frac{1}{N}\sum\limits_{i = 1}^{N} {(y_{i} - \hat{y}_{i} )^{2} } $$

$$ R^{2} = 1 - \frac{{\sum\limits_{i = 1}^{N} {(y_{i} - \hat{y}_{i} )^{2} } }}{{\sum\limits_{i = 1}^{N} {(y_{i} - \overline{y})^{2} } }} $$

Formula $\hat{y}_{i}$ for the model predicted value, $y_{i}$ for the true value.

#### Characteristic of RFR model

The characteristic of the RFR model were set as Table 3.

**Table 3:** Characteristic of RFR model in the proposed methodology.

Characteristic	Value	Function
Number of trees in the forest	100	Refers to the number of decision trees included in the random forest. Increasing the number of decision trees can improve the stability and classification performance of the model, but it will increase computation time. Usually, choosing an appropriate number of decision trees can achieve better results
Number of features to consider when splitting the decision tree each time	$\sqrt {\text{n}}$, n = number of input variables	Refers to the number of features considered when each node performs feature selection. Generally, this parameter needs to be set small to reduce the variance of the model. It is usually recommended to set it to the square root of the total number of features, which ensures that different feature subsets are considered when each decision tree splits, increasing the diversity and generalization performance of the model
Criterion for the split nodes of the decision tree	MSE	Specifies the evaluation criteria for splitting decision tree nodes
Maximum depth of the decision tree	10	Controls the maximum depth that the decision tree can grow. A too large depth can lead to overfitting, while a too small depth can result in underfitting. Therefore, this parameter needs to be adjusted appropriately to achieve the best performance
Minimum number of samples required to split an internal node	5	Controls the minimum number of samples required to split each internal node. If the number of samples in an internal node is less than this value, the node will not generate any child nodes, and the branch at this position will stop growing. Setting this parameter value too large may lead to underfitting, while setting it too small may lead to overfitting
Minimum number of samples required to be at a leaf node	3	Controls the minimum number of samples required for each leaf node. For small datasets, this parameter needs to be set smaller to ensure that the model has enough flexibility

### Materials and methods

#### Structure of SBR

**Figure 2:**
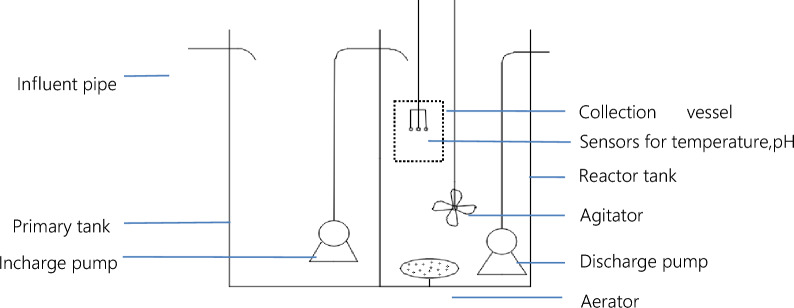
Structure of SBR.

A sequencing batch reactor (SBR), receiving sewage water from a residential area, is prepared in this study. The source of domestic sewage is from the Shuyuan Community in Pidu District, Chengdu, Sichuan, China (longitude: 103.88, latitude: 30.82). The sewage water flowing into the SBR comprised domestic wastewater that had been primary filtered and precipitated. The SBR reactor (stainless steel, 800 mm × 800 mm × 1200 mm) was designed and manufactured. The working volume of the reactor was 0.576 m^3^, respectively (Fig. 2). An agitator and an aeration device are installed in the reaction tank.

Sewage water that had been primary filtered and precipitated was pumped into the SBR. This pump was called pump A which made 0.1856m^3^ sewage fed to the SBR every cycle. Pump B transported the same volume of water out of the SBR when the cycle ended.

#### Control

**Figure 3:**
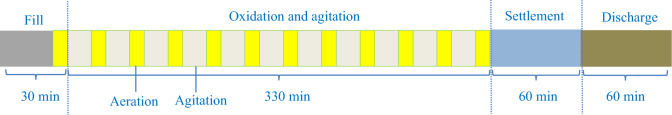
Treatment process of SBR.

The SBR process is automated and controlled by a PIC (Programmable Integrated Circuit) or a one-chip computer. The cycle, which lasts for 480 min, includes the following stages: 30 min fill in and aeration stage, 330 min oxidation and agitation (alternating aeration and agitation, with aeration lasting for 10 min and agitation 20 min) stage, 60 min settlement stage and 60 min discharge stage. Figure 3 shows time management of the operation of SBR.

#### Monitoring

Monitoring influent and effluent wastewater samples were taken from the SBR tank and from a collection vessel which allows filtered water go through in order to get rid of the interference of activated sludge.

Filtered pH and temperature were tested by monitoring sensors which manufactured by LuHeng Co. of China (pH:pH sensor LuHeng 6503; temperature:temperature sensor LuHeng 229).

Filtered COD was tested by potassium dichromate method, NH_3_-N was tested by Nessler’s reagent colorimetry method (SP-756P UV visible photometer of Shanghai spectrum) and TP was tested by spectrophotometric detection method (SP-756P UV visible photometer of Shanghai spectrum).pH and temperature were measured at 10 min intervals by sensors.

Sensors were fitted approximately 200 mm below the lowest liquid level within the reaction tank and above any potential sludge blanket that might be formed during settlement. All instruments were calibrated, maintained and operated in accordance with manufacturer’ instructions.

#### Overview of COD, pH and temperature profiles

**Figure 4:**
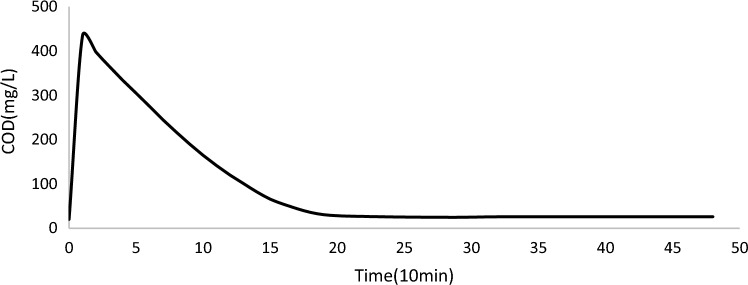
Typical profile for COD.

A typical profile for COD saw an increase in concentrations as influent was mixed with the treated sewage water remaining in the reactor from the previous cycle. COD concentrations peaked soon after the fill phase. Following this peak, COD concentrations decreased due to organic carbon oxidation and nitrification^72^. At approximately 250 min, the rate of decrease in COD concentrations has no more obvious change and continued thus for the rest of the cycle (Fig. 4).

**Figure 5:**
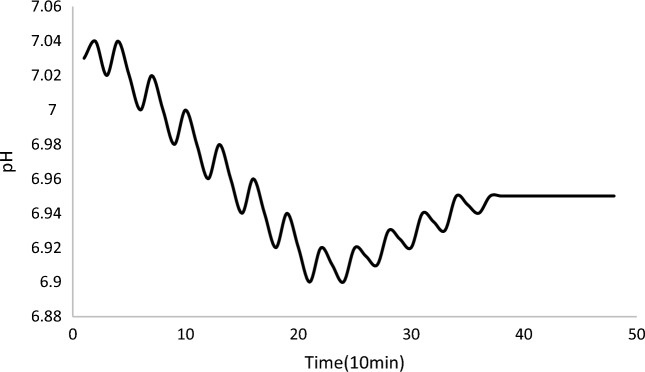
Typical profile for pH.

A cyclical rise and fall in pH (Fig. 5) profile during the aeration phase occurred, as the aerator switched on and off, resulting in a peak and trough in each aeration period in pH profile. The increase in pH, corresponding to the aeration-on period, was likely, in this case, to be due to CO_2_ stripping^73^. The decreases in pH profile during the 20 min stirring period were likely due to a microbial activity which release carbon dioxide^74^.

As found, during the early stage of the cycle, pH fell harder than the later, it is probably because the COD concentration differs: higher COD concentration results in more activity of microorganisms. In general, pH decreases as alkalinity is consumed during the nitrification progress. Denitrification progress causes the overall increase of pH at the medium and end stage probably^75^.

$$ \begin{gathered} {\text{NH}}_{{4}}^{ + } + {\text{2O}}_{{2}} = {\text{NO}}_{{2}}^{ - } + {\text{H}}_{{2}} {\text{O}} + {\text{2H}}^{ + } ({\text{pH decreases period}}) \hfill \\ {\text{NO}}_{{3}}^{ - } + {\text{5H}} = {1}/{\text{2N}}_{{2}} + {\text{2H}}_{{2}} {\text{O}} + {\text{OH}}^{ - } ({\text{pH increases period}}) \hfill \\ {\text{NO}}_{{2}}^{ - } + {\text{3H}} = {1}/{\text{2N}}_{{2}} + {\text{H}}_{{2}} {\text{O}} + {\text{OH}}^{ - } ({\text{pH increases period}}) \hfill \\ \end{gathered} $$

A cyclical rise and fall in pH profiles during the aeration phase occurred, as the aerator switched on and off, resulting in a peak and low-lying valley in each aeration period in pH profiles^76^.

**Figure 6:**
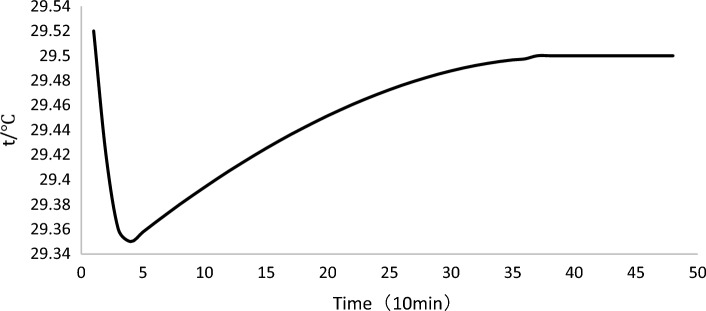
Typical profile for temperature.

A typical profile for temperature descent rapidly as influent was mixed with the treated wastewater remaining in the reactor from the previous cycle. The temperature hit bottom soon after the fill phase. Following this bottom, temperature increased due to microbial activity. At approximately 250 min, the rate of increase in temperature has no more obvious change and continued thus for the rest of the cycle. Figure 6 shows the temperature change in a whole cycle.

As found, during the early stage of the cycle, temperature increased harder than the later, it is probably because the pollutants concentration differs: higher level pollutants concentration results in more activity of microorganisms which is the main reason for temperature change. The overall variation of sewage temperature in a certain cycle is relatively small, which is greatly influenced by the heat conduction and microbial metabolism of the environment, while the mechanical heat transfer, mainly by the pumps and aerators, has little influence on the variation of sewage treatment temperature^77^.

### Application

According to the principle of RFR model, the modeling process is divided into four steps as follows: (1) the collection of sample data; (2) the determination and ranking of the importance of features; (3) different number of features were added to the random forest model in order to select proper quantity of important features; (4) RFR model applied in practice.

**Figure 7:**
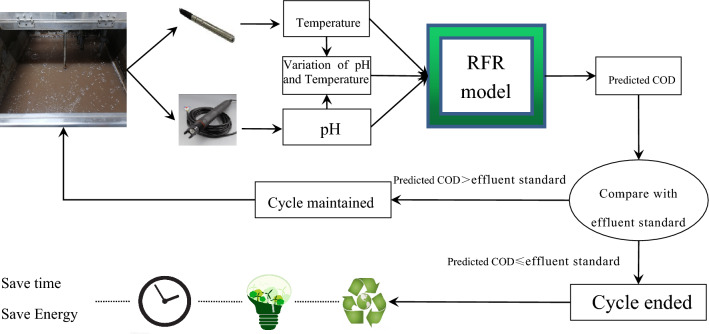
The technical balance between the SBR procedures and RFR algorithm.

Based on the operation process data, RFR soft sensor model is used to establish the COD prediction model of SBR effluent, which realizes the rapid prediction of effluent quality and provides the basis for the efficient and stable operation of the wastewater treatment process as shown in Fig. 7.

#### Assessed Input Variables

In this case, the temperature values was observed to increase with COD reduction and was considered useful in identifying the end of COD removal.

In the early stage of biochemical reaction, the anabolism of microorganism is intense, which produces an amount of CO_2_. The quantity of CO_2_ caused by anabolism is obviously more than that by aeration according to the result of measurement (Fig. 5) in the early stage. Moreover, the organic matter produces organic acid, which makes the pH value decrease further. Less residual organic matter caused lower production of CO_2_ and organic acids, and the predominance of denitrification in the medium and end stage during this time period contribute to an overall increase in pH value. So the pH values were observed to decrease or increase according to different organic matter and was considered useful in identifying the residual quantity of COD.

A number of unprocessed and processed input variables such as pH, temperature, pH and temperature change in adjacent measurements, etc. were constructed and added to the set of independent variables. The selected processed input variables were constructed using the profile features.

The advantage of that pH and temperature were taken as unprocessed variables is it’s simple and easy to detect them. Besides sensors for pH and temperature are not only low-cost but also have satisfying measurement accuracy.

Each variable set included a unique collection of input variables (Table 4). Within each 480 min cycle, data collected 0 ~ 30 min and 361 ~ 480 min were excluded to eliminate the effects of filling and settlement periods (as these phases were not part of the biological reaction phases of the treatment cycle).

**Table 4:** Input variables.

Input variables	Description
pH	Raw pH data
∆pH	∆pH = pH_i_ − pH_i-1_, the difference between current pH value and previous
pH_av_	Moving average of pH over the previous 10 records
pH_apex-nadir_	pH apex value minus pH nadir value for each aeration period
T	Raw temperature data
∆T	∆T = T_i_–T_i-1_, the difference between current t value and previous
T_av_	Moving average of T over the previous 10 records
pH·T	pH multiply by T
pH_av_·T_av_	pH_av_ multiply by T_av_
T/pH	T divided by pH
T_av_/pH_av_	T_av_ divided by pH_av_
∆T·∆pH	∆T multiply by ∆pH

Data from 40 treatment cycles were collected, 12 (30%) of which were randomly separated for use as a test dataset, and the remainder were used as a training dataset.

#### Assessment of RFR soft sensor model

The effectiveness of the RFR soft sensor model was assessed across 5 criteria. The effluent standard value of COD was set at 30 mg/l. Effluent standard value can vary due to local regulations. The assessment criteria are listed in Table 5.

**Table 5:** Criteria of assessment.

Criterion	Description	Practical application
R^2^	Referred to as the coefficient of determination, it is an indicator of the strength of the relationship between variables	Measures the strength of the relationship between predicted COD trend and actual trend
MSE	Mean square error (MSE) is a standard statistical metric to measure model performance; it measures the difference between sample and predict values and is a good measure of accuracy. The lower the MSE value the more accurate the prediction	Measures the average accuracy of the predicted COD trend against the actual trend
Percentage of COD removal	This criterion returns the percentage COD removal from the peak true concentration of the measured treatment cycle (30 ~ 360 min) to the predicted COD concentration which is below the effluent standard value for the first time	Provides a comparison of the COD concentration at which the cycle would have been ended by the model during a controlled cycle and the COD peak concentration at the beginning of a cycle
Percentage of time saved (T_save_)	T_save_ = (330 − T_thres_)/330 where T_save_ is the time saving (%), T_thres_ is the time at which the cycle would be ended by the model in a controlled scenario and 330 is the fixed time cycle length (min) set in an uncontrolled scenario	Indicates the time saved with the selected cut-off threshold value In general, the greater the time saved, the more the energy saved only if the accuracy is met the requirement
Accuracy	When the predicted COD concentration is below the effluent standard value for the first time, if it is true (predicted COD > measured COD) the accuracy meets the requirement. Symbol “ + ” for meeting the requirements, otherwise “ − ”	Indicates the accuracy at the cut-off threshold value

## Results

### Average influent and effluent results

Table 6 shows the related parameters of influent and effluent.

**Table 6:** Average influent and effluent results.

Parameters	Average influent (mg/L)	Average effluent (mg/L)	Average removal (%)
COD	305	23	92.46
TP	0.97	0.10	89.69
NH_3_-N	20.6	1.1	94.66

### Ranking of variables

Pearson correlation coefficient is a statistical measure used to determine the strength and direction of the linear relationship between two variables. Essentials of application of Pearson correlation coefficient in variables correlation ranking are: (1) Pearson correlation coefficient is commonly used in multiple regression analysis to select the most significant independent variables by calculating the correlation coefficients between each independent variable; (2) The correlation coefficient ranges from − 1 to 1, and the larger the absolute value, the stronger the correlation; (3) When the correlation coefficient value is close to 0, it indicates that the correlation between the two variables is very weak and they can be considered independent.

**Figure 8:**
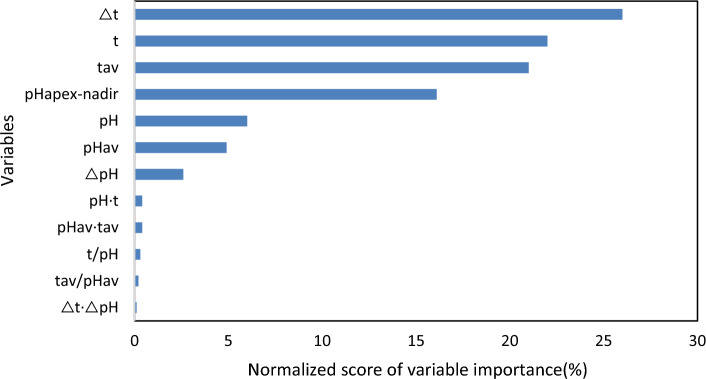
Ranking of each variable importance score.

In order to guarantee the training effect of the RFR, the Pearson coefficient method was used to study the correlation of the subjects, and the variables with weak correlation were deleted. In order to prevent the occurrence of invalid variables, avoid overfitting and improve the training performance of the model, any variable with a normalized Pearson correlation coefficient value, that is regarded as the normalized score of variable importance, less than 0.01 was removed. The resulting normalized score of variable importance ordering diagram shows the 12 factors affecting COD concentration (Fig. 8). It was found that ΔT had the greatest influence on COD concentration, followed by T, T_av_, pH_apex-nadir_ and etc.

Data from 40 treatment cycles were collected. 70% of the whole data (28 treatment cycles) are randomly selected as training set for RFR model and 30% (12 treatment cycles) are selected as testing set to verify the accuracy of the model.

Based on the ranking of variable importance scores, it is evident that temperature-related variables hold the top three positions. Therefore, it can be concluded that temperature-related variables play a dominant role in the data analysis. Some studies have shown that the metabolic activity of microbial communities in wastewater treatment bioreactors can cause an increase in water temperature^78,79^. This is because the microorganisms in the reactor produce a large amount of heat through the degradation and metabolism of organic matter, leading to an increase in the temperature inside the reactor. Furthermore, it should be noted that while pH is indeed a contributing factor, its significance is not as strong as that of pHapex-nadir. pHapex-nadir, which is calculated by pH apex value minus pH nadir value for each aeration period, effectively quantifies the amount of carbon dioxide generated by microbial activity during a 20 min agitation.

### Variables definition

**Figure 9:**
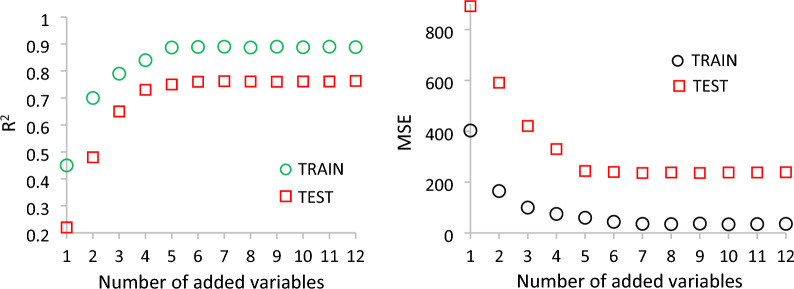
Evaluation indexes with different quantitative of variables.

In order to select the variables set, different numbers of variables were selected according to the importance of variables, and then were added to the RFR model, as shown in Fig. 9. It was found that when the top 7 variables were selected, the R^2^ of training set and the test set did not increase and the MSE did not decrease obviously, so the top 7 variables were selected as the variable of the optimized RF model, specific as follows: ∆T, T, Tav, pHapex-nadir, pH, pHav and ∆pH.

### Predict results by RFR

**Figure 10:**
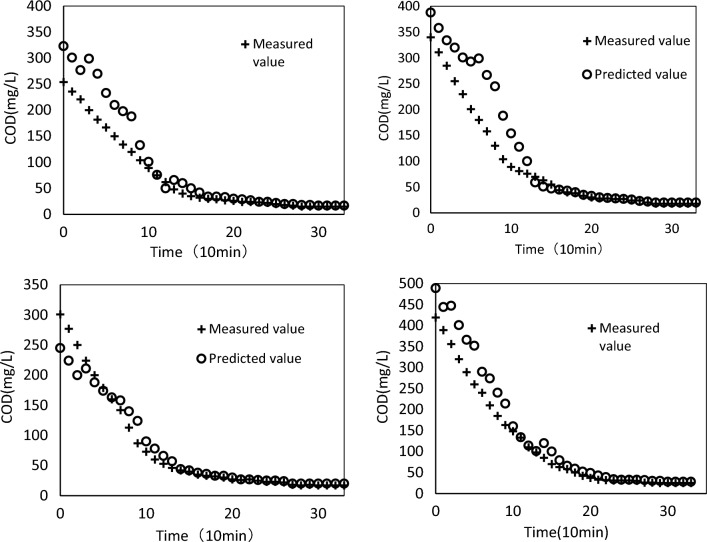
Comparison of predicted and actual/measured COD concentrations.

Figure 10 shows the comparison of predicted and measured COD concentrations on the test set.

The COD degradation trend, as well as the deviation between predicted and measured values, can be observed from the variations in the curves depicted in Fig. 10. The predicted values enable a rough estimation of the processing effect and level of pollutant degradation within a single cycle. Although the accuracy between true and predicted values may not be perfect, the slight discrepancy only exists in the initial stage of the process and soon disappears.

In the process of sewage treatment, the change of COD is influenced by various uncertain factors in operating conditions. These factors can cause significant differences in the accuracy of prediction of COD during different stages of processing. In the early stage, these uncertain factors have a stronger influence, which results in a obvious error between the predicted and measured values; with the passage of time, the processing conditions tend to stabilize, and the impact of uncertain factors on COD changes gradually decreases, leading to a reduction in the error between predicted and measured values.

Therefore, in the proposed methodology, the magnitude of the error between predicted and measured values is mainly affected by the processing stage. In the early stage, the error may be relatively large, but as time progresses, the error will gradually decrease and eventually reach a more accurate prediction effect.

**Figure 11:**
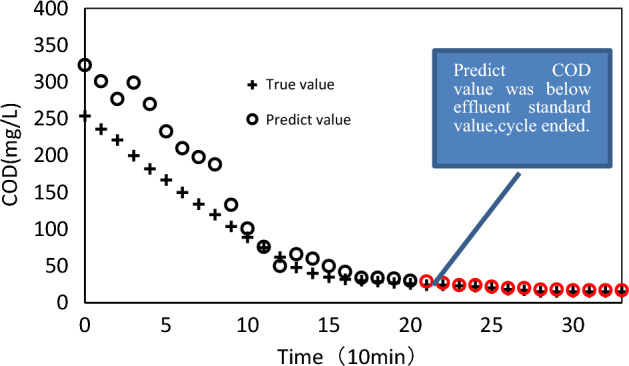
Condition of cycle ends.

The RFR soft sensor model output, serving as the predicted value of water quality in the given scenario, can be instrumental in optimizing the wastewater treatment process. This can be achieved by reducing energy consumption and enhancing the efficiency of chemical and biological processes. Specifically, if the predicted COD value falls below the effluent standard level, the process can transition into settlement mode immediately, with the agitator and aerator being switched off, thus bringing the cycle to a close. This method allows for cycle completion to be controlled by an artificial intelligence and automatic control system, as opposed to a fixed-time control approach that lacks precision. This is illustrated in Fig. 11.

Table 7 shows the assessment results of the test set 1–12.

**Table 7:** Results of assessment.

Criterion	Test set												Average
	1	2	3	4	5	6	7	8	9	10	11	12	
R^2^	0.77	0.7	0.72	0.79	0.76	0.75	0.84	0.77	0.77	0.71	0.78	0.79	0.763
MSE	264	268	245	247	218	197	199	235	270	265	240	216	239
Percentage of COD removal (%)	88.6	91.5	91	93.4	90	87.7	88.1	93.8	92.1	90.4	92.7	93.6	91.075
Percentage of time saved (%)	36.4	33.3	36.4	9.1	36.4	39.4	33.3	6.1	12.1	18.2	18.2	12.1	24.25
Accuracy	+	+	+	+	+	−	+	+	+	+	+	+	Percentage of hits 91%

## Discussion

### Benefits of the RFR soft sensor model

RFR is a machine learning model used for predictive analytics tasks, particularly for regression problems. RFR is an ensemble learning method that combines multiple decision tree models to create a more robust and accurate predictor.

The RFR algorithm randomly selects subsets of the input variables and samples of the training data to construct decision trees, which are then combined into a forest. During prediction, the RFR model aggregates the output of individual decision trees to produce a final prediction. This approach helps to reduce the impact of overfitting and improves the model's performance on output data.

In the context of wastewater treatment plants, RFR *soft sensor* model can be used to predict water quality (COD) through simpler diameters, in this way complex and expensive sensors will be replaced. Although a genuine COD sensor may be an option, several reasons or factors can result in its unsuitability and cause certain issues to arise: (1) Genuine COD sensors usually cost much; (2) Due to the presence of suspended solids in sewage, the COD value measured by genuine COD sensors can be unstable and exhibit significant fluctuations; (3) Some genuine COD sensors can detect organic compounds with a double bond sensitively while other organic compounds without a double bond are failed to be detected, so the error can not be ignored.

Moreover, the RFR model overcomes the shortcomings of slow convergence speed and large number of samples requiring of neural networks. Neural networks are powerful models that can learn complex patterns in data. However, training a neural network can be computationally expensive and require a large amount of data. In particular, deep neural networks or large-scale networks may take a long time to converge during training due to the sheer number of parameters that need to be learned through multiple iterations.

In contrast, RFR model are composed of multiple decision tree models, each trained on a random subset of the data. This approach has advantages: (1) RFR model does not require as much data as neural networks, since each decision tree model can work well with smaller datasets; (2) RFR model can be easily parallelized, which means they can be trained more quickly than neural networks on multi-core computer systems.

### Comparison RFR soft sensor model with others

Comparing with methods used in pollutant removal technology (Table 1), the proposed methodology requires only two types of raw data that are easy to obtain, greatly reducing the workload of data acquisition. The weakness of the proposed methodology is that prediction value is not so accurate between measured and predicted value at the first stage of the progress. Additionally, even if the R^2^ and MSE of RFR model are not satisfactory, it performs well in predicting accuracy at the cut-off threshold value, as shown in Table 7, and this is very concerned in the field of engineering.

### Potential trade-offs or unintended consequences

In the practical application of the proposed methodology, it is possible that the COD value meet the standard while other indicators such as ammonia or phosphorus do not. To address this issue, relationship models can be established using pH and temperature as variables to predict the other parameters. However, this approach is limited to artificial intelligence methods only. In addition, an empirical judgment system can be established, such as the sewage treatment time generally being within a certain range, if predicted results exceeds this range, the output results of the proposed methodology are deemed to require modification.

### Methods or options for improvement

Increase conductivity or other readily available parameters as input variables to improve prediction accuracy. The following are detailed discussions:

Variables such as conductivity, MLSS, DO and ammonia can also be used as premises to predict COD. The impact to the accuracy and efficiency of the proposed methodology may be: (1) In general, in the sewage treatment process, the electrical conductivity of the solution shows a trend of decreasing gradually, which is related to the decrease of COD value, hence the electrical conductivity may improve the accuracy and efficiency; (2) MLSS should show a trend of increasing gradually, however, the change of MLSS is not obvious in one cycle (480 min). Furthermore, the accuracy of MLSS sensor is easily affected by the color of wastewater, this will obviously increase the uncertainty of the data measured by the sensors; (3) The SBR works according to aeration-agitation periodicity, DO presents increase–decrease periodicity change, which obviously has no correlation with COD value change trend; (4) During the sewage treatment process, the ammonia concentration in the solution generally exhibits a gradual decrease, similar to the trend observed in COD. However, in some cases, such as a lack of dissolved oxygen that inhibits nitrification, there may be no significant reduction in the ammonia value even when COD is reduced. As a result of the non-synchronous nature of the changes in these two parameters, predicting COD using ammonia as a variable may introduce uncertainty into the analysis.

Encrypt the frequency of data acquisition, such as collecting data every 5 minutes, then it can be five minutes in advance to predict, which further improves the efficiency of the proposed methodology.

Add ammonia and phosphorus as prediction targets to balance organic and inorganic wastewater indicators and improve practicality.

## Conclusions and outlook

Simple and stable sensors (pH, temperature) were utilized to predict COD values throughout the process. The RFR model employed in the study can be regarded as a "soft sensor", which assists in monitoring the treatment effect.

The SBR was optimized using artificial intelligence and an automatic control system to increase automation, as well as save both time and energy. pH and temperature sensors collected data, which were input into the RFR model, the model then outputted real-time COD values. Once the predicted COD value fell below the effluent standard value, the cycle ended by cutting down the agitator and aerator, and the process entered the settlement mode directly. The proposed methodology replaced fixed-time control, which was uncontrolled. In 12 test cases, the percentage of COD removal (%) was about 91. 075, while an average of 24. 25% of time or energy was saved. These results demonstrate that this approach can increase treatment capacity and reduce energy consumption, representing a low-carbon technology.

R^2^ on the test set is around 0.791, although it is not too high, but the accuracy at the cut-off threshold value of COD is around 91% which is acceptable for the prediction. It is quite simple and almost accurate to acquire the processing effect and the level of degradation of pollutants at anytime. Although it is not so accurate between true and predict value, but the embarrassment only occured at the first half of the progress and it soon vanished. The accuracy of the medium and end stage is more important than that of the early stage, the reason for the above fact is explained below. Artificial intelligence and automatic control system leaded to a optimized way but the satisfied accuracy of predict COD value is prerequisite. Basing on the fact, accuracy requirements are different at each stage in a controlled scenario: in the medium and end stage, especially when approaching the stage of effluent standard compliance, greater emphasis is placed on precision and accuracy. However, in the early stage, the accuracy does not significantly affect the control strategy.

Due to the non-linearity and uncertainty of the variation of pH value with time in SBR process, predict results are unstable because of different algorithm and over-fitting by ANN method. Due to the parallel information distribution and storage of structural preprocessing, RFR has strong fault tolerance and the ability to adapt to the external environment through learning. The ability of pattern recognition and comprehensive reasoning undoubtedly opens up a broad prospect for experimental research.

One limitation of this research is its exclusive focus on SBR methodology. However, there exists the potential to modify the procedure to cater to other technologies, particularly batch wastewater treatment systems. By increasing the frequency of data acquisition, such as collecting data every 5 minutes, it may be possible to predict factors up to five minutes ahead of time, thereby further enhancing the efficiency of the proposed methodology. To improve its practicality, ammonia and phosphorus could be included as prediction targets, as this would help balance organic and inorganic wastewater indicators.

## Data Availability

The datasets used and/or analysed during the current study available from the corresponding author on reasonable request.
